# Auditory Motion Elicits a Visual Motion Aftereffect

**DOI:** 10.3389/fnins.2016.00559

**Published:** 2016-12-05

**Authors:** Christopher C. Berger, H. Henrik Ehrsson

**Affiliations:** Department of Neuroscience, Karolinska InstitutetStockholm, Sweden

**Keywords:** visual motion aftereffect, auditory perception, multisensory perception, auditory motion, visual motion perception

## Abstract

The visual motion aftereffect is a visual illusion in which exposure to continuous motion in one direction leads to a subsequent illusion of visual motion in the opposite direction. Previous findings have been mixed with regard to whether this visual illusion can be induced cross-modally by auditory stimuli. Based on research on multisensory perception demonstrating the profound influence auditory perception can have on the interpretation and perceived motion of visual stimuli, we hypothesized that exposure to auditory stimuli with strong directional motion cues should induce a visual motion aftereffect. Here, we demonstrate that horizontally moving auditory stimuli induced a significant visual motion aftereffect—an effect that was driven primarily by a change in visual motion perception following exposure to leftward moving auditory stimuli. This finding is consistent with the notion that visual and auditory motion perception rely on at least partially overlapping neural substrates.

## Introduction

The visual motion aftereffect (MAE) is a well-known visual illusion in which exposure to a continuously moving visual stimulus results in the subsequent illusory perception that a static image moves in the opposite direction. The classic example of this is if one looks at a waterfall for a period of time and then looks at the stationary rocks to the side of the waterfall, the rocks appear to be moving upwards—also known as the “waterfall illusion” (Anstis et al., 1998). It is largely accepted that this visual illusion originates in the visual cortex (Tootell et al., 1995; He et al., 1998; Taylor et al., 2000; Huk et al., 2001; Hogendoorn and Verstraten, 2013) and involves the adaptation of motion sensitive neurons in the medial temporal area of the visual cortex known as area MT or V5. However, cross-modal interactions and influences on visual perception are relatively common (Shams et al., 2000; Shimojo and Shams, 2001; Hidaka et al., 2011b), and research investigating motion perception in a multisensory context has indicated that MAEs can also be induced cross-modally. For instance, Konkle et al. (2009) observed that tactile stimulation delivered to the hand could induce a visual MAE, suggesting that the processing of visual and tactile motion rely on shared neural representations that dynamically impact modality-specific perception (Konkle et al., 2009). In the auditory domain, however, findings from research investigating whether cross-modal MAEs could be obtained from auditory stimuli have been unclear.

In investigating whether auditory motion stimuli can induce MAEs, Kitagawa and Ichihara (2002) found that although visual motion could alter the subsequent perception of looming or receding auditory stimuli (as indicated by increases or decreases in loudness, respectively), looming or receding auditory stimuli did not elicit a visual MAE. Kitagawa and Ichihara (2002) concluded that this finding supports the notion of visual dominance over auditory perception for spatial events. This finding is in contrast, however, with those of Maeda et al. (2004) who found that auditory stimuli consisting of sounds increasing or decreasing in pitch was sufficient to induce cross-modal changes in visual motion perception in the vertical plane (Maeda et al., 2004), as well as findings by Hedger et al. (2013) who found that music containing ascending or descending scales can induce visual MAEs in the vertical plane (Hedger et al., 2013). However, these studies rely on the metaphorical relationship between a sound and an associated visual representation rather than on physical motion cues of the sounds themselves. In this way, the findings of Maeda et al. (2004) and Hedger et al. (2013) are much like the positive aftereffects (i.e., changes in visual motion perception in the same direction as the adapting auditory stimulus) found in the sound contingent visual motion aftereffect (Hidaka et al., 2011a), and the negative aftereffects (i.e., changes in visual motion perception in the opposite direction as the adapting auditory stimulus) found for verbal language descriptions of visual events (Dils and Boroditsky, 2010), respectively. Furthermore, the looming/receding auditory motion stimuli used in Kitagawa and Ichihara's (2002) study were simply tones increasing or decreasing in volume and provided weak motion cues compared to those from veridical horizontal auditory motion (Carlile and Leung, 2016). Thus, although previous studies have been mixed as to whether auditory motion is sufficient to induce a visual MAE, none of the studies to date have examined whether visual MAEs can occur following adaption to auditory stimuli with horizontal apparent motion cues.

Here, in a very simple experiment, we sought to examine whether an arbitrary (i.e., a sound with no metaphorical visual association) auditory stimulus (440 Hz sine-wave tone) with horizontal motion cues could induce a visual motion aftereffect. To this end, we created custom binaural recordings of a 440 Hz sine-wave tone moving horizontally in front of the participant from right-to-left (leftward adaptation) or left-to-right (i.e., rightward adaptation) and measured the participants' motion perception using a standard motion discrimination task commonly employed to examine visual MAEs (Hiris and Blake, 1992; Blake and Hiris, 1993). We hypothesized that if auditory motion can induce a visual MAE, then we should observe a significant difference between the motion coherence levels at which the participants can no longer distinguish between a leftward moving display and a rightward moving display—i.e., the points of subjective equality (PSE) for motion discrimination—following exposure to leftward vs. rightward auditory motion. Specifically, we hypothesized that this shift in the PSEs would be gleaned from an increased propensity to perceive a motion display as moving rightward following leftward sound adaptation compared to following rightward sound adaptation and an increased propensity to perceive a motion display as moving leftward following rightward sound adaption.

## Methods

### Participants

Fifteen participants were recruited to participate in this experiment. All participants were recruited from the student population in the Stockholm area, were healthy, reported no history of psychiatric illness or neurologic disorder, and reported no problems with hearing or vision (or had corrected-to-normal vision). All participants provided written informed consent before the start of the experiment. The study was approved by and conducted in accordance with the Regional Ethical Review board of Stockholm.

### Auditory stimuli

The auditory stimuli consisted of a binaural recording of a 440 Hz sine-wave tone “sweeping” horizontally from left to right in front of the dummy head with a studio-quality microphone inside each ear canal of a dummy head (KU 100 dummy head audio system; Neumann artificial head stereo microphone). The auditory sweeps spanned approximately 150 cm in the x-plane (beginning and ending 75 cm to the left and right of the dummy head), with a distance of ~40 cm in front of the dummy head in the y-plane, and 0 cm displacement in the z-plane relative to the ears of the dummy head. From this initial recording, an auditory motion stimulus was generated using the audio editing software Audacity® (version 2.0.6). Each leftward moving sweep was 456 ms in duration, with 22 ms of silence added to the onset and offset of each sweep. Additionally, 500 ms of silence was added at the very beginning and after every ninth sweep. Initial piloting revealed that the addition of a silent break after every ninth sweep was sufficient to prevent the perception that the sounds were oscillating rather than moving left to right. The rightward moving auditory stimulus was created by switching the left and right stereo tracks. The auditory motion stimuli were presented to the participants during the experiment using in-ear headphones.

### Visual motion stimuli

The visual motion test stimuli consisted of low-contrast dynamic random dot kinematograms. The dot displays consisted of 100 dots, each with a diameter of 0.12° moving in a circular window (diameter = 15.19°). The dots moved at a speed of 99.26°/s. The coherence of the dots (i.e., the percentage of dots moving in the same direction) was manipulated so that a specified percentage of the dots were moving to the left or to the right in leftward motion and rightward motion test displays, respectively. That is, for each test stimulus, a particular percentage of the dots moved coherently to the left or to the right, whereas the remaining dots moved randomly for the duration (1 s) of the visual motion test stimulus.

### Baseline motion sensitivity assessment

During the baseline motion sensitivity assessment, the participants were instructed to maintain fixation on a light-gray central fixation dot (diameter = 0.19°) on a 21.5-inch (54.61 cm) iMac computer screen (viewing distance = 60 cm) with their head in a chin-rest while motion test stimuli were presented to them. The participants then made leftward or rightward forced-choice judgments about the direction of motion of test stimuli by pressing the “left” or “right” keys on the keyboard, respectively. The baseline motion sensitivity task consisted of 192 test stimuli for rightward and leftward motion (96 test stimuli per direction) with the following 12 coherence levels: 99, 66, 44, 29, 20, 13, 9, 6, 4, 3, 2, and 1%. Thus, each coherence level was repeated 8 times for each motion direction (i.e., leftward or rightward). Immediately following the end of the baseline task, the participants' data were fitted with a logistic regression function. From this fit, each participant's accuracy threshold for each direction was calculated (i.e., the coherence value at which the participant achieved 90% accuracy for leftward moving stimuli and the coherence value at which the participant achieved 90% accuracy for rightward moving stimuli). These coherence values, and coherence values corresponding to half (i.e., 50%) and one-quarter (i.e., 25%) of these values, for rightward and leftward motion, were then used as the coherence levels of test stimuli in the main experiment (i.e., 6 coherence values; 3 per direction). These participant-specific coherence values were subsequently coded as normalized coherence values and are referred to in the results as normalized coherence values. If the participants' performance was so poor that their 90% coherence threshold value was calculated to be >100% coherence for a given direction (i.e., if more than 100% of the dots would need to be moving in a specific direction for the participant to achieve a 90% accuracy in the motion discrimination task for either direction based on their baseline performance), then that participant did not participate in the subsequent auditory adaptation experiment. Three of the fifteen recruited participants were excluded from the adaptation portion of the experiment for this reason.

### Auditory motion adaptation procedure

**Figure 1:**
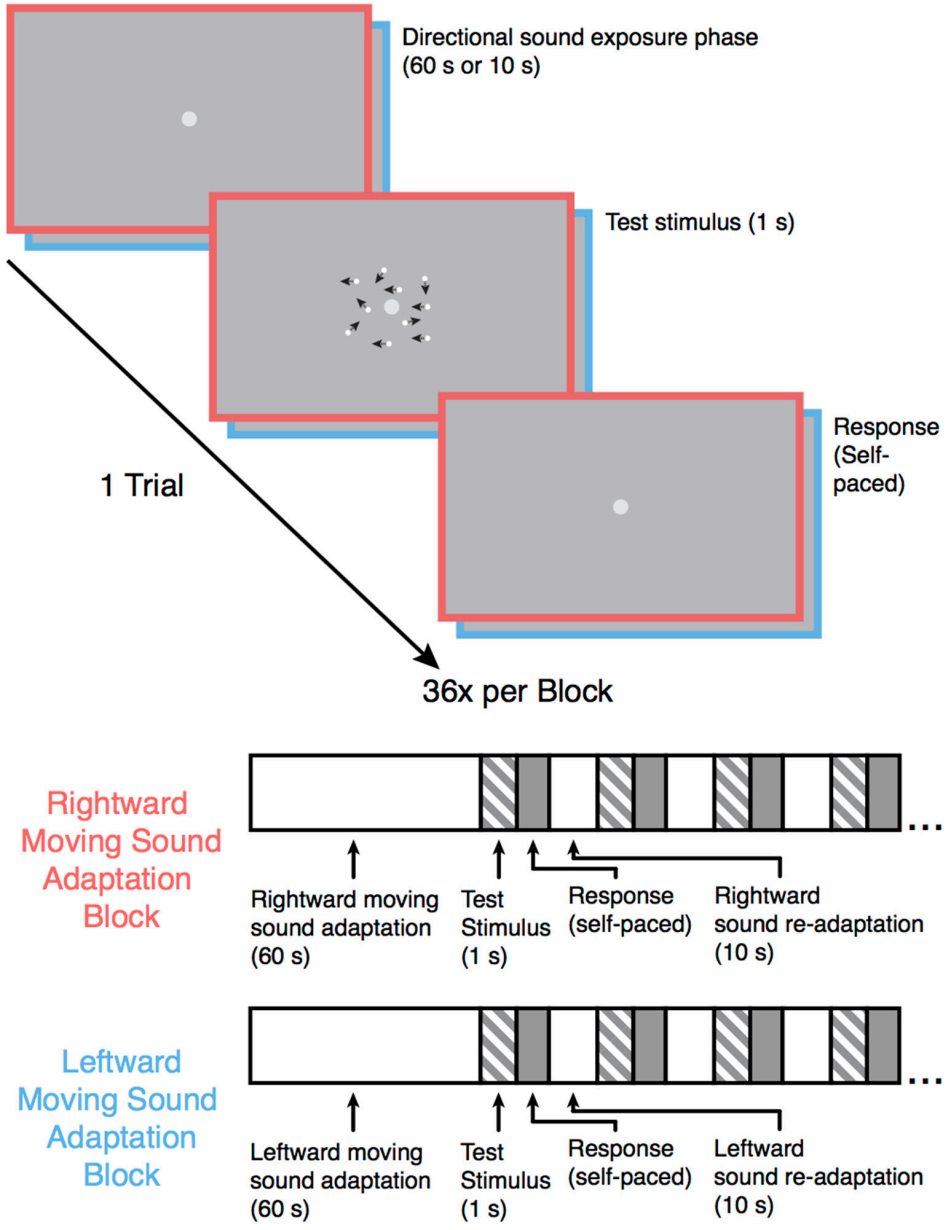
**Schematic overview of the experiment**. Following the baseline motion sensitivity assessment, the participants began one of the auditory motion adaptation (leftward or rightward) blocks. Participants were instructed to maintain fixation on the central fixation point during the experiment. Each trial began with an auditory motion exposure phase (60 s for the first trial and 10 s for each subsequent trial in that block) followed by one of six possible test motion stimuli lasting 1 s (the coherence level for each of the 6 motion stimuli was individually catered to each participants' baseline performance), and the participants subsequently indicated whether they saw the test stimulus move leftward or rightward. The test stimulus in the above example trial shows a 50% coherence trial for illustration purposes. The black arrows in the example test stimulus indicate the direction of each dot in this example for display purposes only. Each sound motion adaptation block was followed by a sound motion adaptation block with the sound moving in the opposite direction and was repeated once, resulting in 4 blocks total. Block order was counterbalanced across participants.

Following the baseline motion discrimination assessment, the participants were once again instructed to maintain fixation on the light-gray central fixation dot while they performed the motion-discrimination task again but with the participant-specific motion coherence values. In each trial, the participants were exposed to an auditory motion stimulus prior to each test stimulus (see Figure 1). The experiment was split into four blocks (36 trials each; 6 repetitions of each of the 6 participant-specific coherence values). Two of the blocks were leftward sound adaptation blocks in which the leftward moving auditory stimulus was presented prior to each visual motion test stimulus, and the other two blocks were rightward sound adaption blocks in which the rightward moving auditory stimulus was presented prior to each visual stimulus. Each auditory motion adaption block was followed by the converse auditory motion adaption block (i.e., a rightward sound adaption block always came after a leftward sound adaptation block and vice versa), and the order was counterbalanced across participants. In each block, the first auditory motion stimulus was presented for 60 s, with each subsequent auditory motion stimulus within a block presented for 10 s. This “top-up” method of adaption is commonly employed in MAE studies (Dils and Boroditsky, 2010; Winawer et al., 2010; Hedger et al., 2013) and was used here to ensure that any established adaptation would not diminish before the end of the block. Stimulus presentation and data acquisition were controlled using PsychoPy (Peirce, 2007) software (version 1.82.01) on a 21.5-inch (54.61 cm) iMac computer, and the experiments took place in a soundproof testing room (40-decibel noise reduction).

## Results

**Figure 2:**
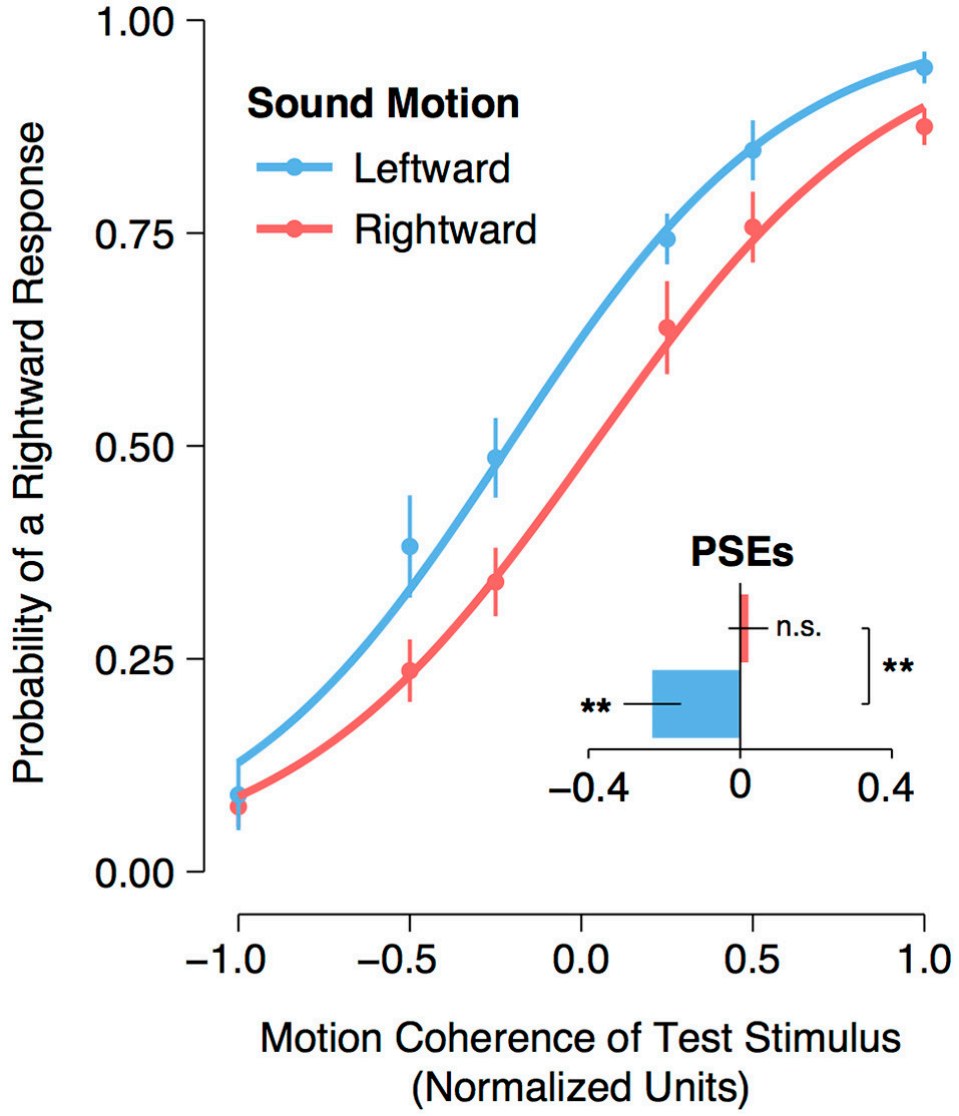
**Visual motion aftereffect following leftward and rightward auditory motion**. Curves represent logistic regression functions fitted to group data. The data points represent the mean frequency of a “rightward” response. Normalized coherence values are represented on the x-axis, with negative values arbitrarily assigned to leftward moving motion displays and positive values assigned to rightward moving displays. The bar plot represents the participants' mean point of subjective equality (PSE) for the leftward and rightward auditory motion adaptation conditions. Asterisks next to bars indicate a significant (*p* < 0.01) shift in the participants' PSE compared to a normalized coherence test value of zero, and “n.s.” indicates that there was no significant shift (*p* > 0.05) from zero. Asterisks between bars indicate a significant (*p* < 0.01) difference between the participants' PSEs for the rightward and leftward auditory motion adaptation conditions. Error bars represent ± SEMs.

A logistic regression was fitted to each participant's motion detection data for each sound direction condition (leftward motion or rightward motion). From each participant's individual model fit, the point of subjective equality (PSE) (i.e., the normalized coherence level for which participants could no longer distinguish whether the dots were moving leftward or rightward) was calculated as the normalized coherence level for which the probability of making a rightward response was 50%. An assessment of the logistic regression function fitted to the group data with a single predictor variable (i.e., the normalized coherence values) confirmed that the model fit was significantly better than the null model for both the leftward [$\chi_{\left(1\right)}^{2}$ = 341.00, *p* < 0.001] and rightward [$\chi_{\left(1\right)}^{2}$ = 319.26, *p* < 0.001] adaptation conditions. Moreover, a planned comparison of the participants' PSEs revealed a significant [*t*_(11)_ = 3.16, *p* = 0.009] shift leftward of the participants' PSEs following exposure to a leftward moving sound (*M* = −0.232, 95% CI [−0.378, −0.084]) compared to the participants' PSEs following exposure to a rightward moving sound (*M* = 0.021, 95% CI [−0.081, 0.124]). One-sample *t*-tests comparing the participants' PSEs following leftward and rightward adaptation to the baseline motion coherence level (i.e., 0) revealed a significant shift leftward [*t*_(11)_ = −3.07, *p* = 0.005] following leftward adaptation but no significant shift to the right following rightward adaptation [*t*_(11)_ = 0.41, *p* = 0.35] (see Figure 2). All analyses and statistical tests were performed using the statistical software program R (R Core Team, 2016).

## Discussion

In this experiment, we demonstrated that a moving auditory stimulus can lead to a visual motion aftereffect. Specifically, we found that listening to an auditory stimulus moving in a specific direction prior to the presentation of a visual stimulus increased the probability that one perceived the visual stimulus as moving in the opposite direction relative to the preceding auditory stimulus. To the best of our knowledge, this finding is the first to clearly establish that motion cues from auditory stimuli alone can lead to a visual motion aftereffect. Moreover, our finding settles an unresolved question in the multisensory literature concerning whether auditory motion can produce visual MAEs.

Previous studies investigating whether auditory stimuli can elicit visual MAEs have either made use of auditory stimuli containing weak spatial cues (i.e., increasing or decreasing loudness; Kitagawa and Ichihara, 2002) or have relied on sounds that elicit strong visual motion metaphors (Maeda et al., 2004; Hedger et al., 2013) or were strongly associated with visual-motion stimuli (Dils and Boroditsky, 2010; Hidaka et al., 2011a). Thus, the results from previous studies have led to somewhat mixed conclusions about whether a cross-modal MAE from auditory stimuli is possible. Here, we specifically examined whether auditory motion signals from an arbitrary (i.e., sound with no associated visual motion metaphor) sound could induce a visual MAE and found that, indeed, apparent auditory motion is sufficient to elicit a visual MAE. Additionally, our finding seems to be at odds with those of Kitagawa and Ichihara (2002) which suggest that visual MAEs cannot occur from auditory stimuli. We speculate that in Kitagawa and Ichihara's failure to observe a visual MAE from looming and receding auditory stimuli is due to the poor spatial cues provided by looming and receding auditory stimuli compared to those of horizontally moving auditory stimuli (Carlile and Leung, 2016).

This interpretation of our finding is consistent with the maximum likelihood estimation view of multisensory perception (Ernst and Banks, 2002; De Gelder and Bertelson, 2003; Ernst and Bülthoff, 2004) as well as findings in research on multisensory integration at large demonstrating that auditory stimuli can have a profound influence on visual perception if the auditory stimuli are sufficiently reliable (Driver and Spence, 2000; Shimojo and Shams, 2001; Ghazanfar and Schroeder, 2006; Alink et al., 2012). In the case of motion perception in particular, for example, it has been demonstrated that auditory spatial information provided by alternating sound locations can cause a static visual stimulus to be perceived as moving in an illusion referred to as the sound-induced visual motion illusion (Hidaka et al., 2011b) and that auditory motion stimuli can capture ambiguous visual motion and change the perceived apparent motion of visual stimuli in the same direction as the moving auditory stimuli (Alink et al., 2012). Furthermore, functional magnetic resonance imagining (fMRI) experiments have revealed that visual motion area MT/V5 responded to auditory motion in blind patients (Saenz et al., 2008), that visual motion capture of auditory motion is reflected in increased activity in area MT/V5 (Alink et al., 2008), and that concordant audiovisual motion stimuli lead to enhanced activity in area MT/V5 compared unimodal motion stimuli or discordant audiovisual motion stimuli (Scheef et al., 2009). Thus, together with previous findings, our finding suggests that visual and auditory motion perception rely on shared neural representations that dynamically impact modality-specific perception.

Although, our primary interest in this study was concerned with whether there were clear differences in motion discrimination following leftward and rightward adaptation, it is interesting to note that our results appear to indicate a stronger shift leftward in the PSE following leftward adaptation as the mean PSE for rightward adaptation is near zero. This finding is in contrast to previous findings demonstrating that rightward moving auditory stimuli could capture ambiguous visual motion stimuli, but that leftward moving stimuli did not (Alink et al., 2012). In our study, we made use of pre-recorded leftward moving auditory motion stimuli that were digitally reversed so that they were perceived as moving rightward. Although, this procedure held the timing and quality of the auditory motion stimuli constant, we cannot rule out that this could have led to some unforeseen differences between the leftward moving and rightward moving stimuli that could have somehow affected the vMAE. However, this interpretation is incompatible with the opposite asymmetry found in Alink et al.'s (2012) study in which an identical presentation protocol was used to present the leftward and rightward auditory motion stimuli from external speakers but only an effect of rightward auditory motion on visual motion perception was observed. One alternative possibility that reconciles the findings of Alink et al. (2012) and ours is that that the influence of auditory stimuli on visual motion perception is anisotropic (i.e., not equal for movement in both directions) as both findings indicate an increase in the perception that ambiguous visual motion stimuli are moving rightward but not that they are moving leftward. However, this interpretation would be at odds with the existing literature on visual motion perception and visual motion aftereffects which largely suggest that the motion processing mechanisms of centrally-viewed motion stimuli in the horizontal plane are isotropic (Levinson and Sekuler, 1975; Ball and Sekuler, 1979; Mather, 1980; Raymond, 1993). Thus, future research may serve to examine whether the observed asymmetry of the effect of auditory stimuli on ambiguous visual motion perception observed here and in Alink et al.'s (2012) study represents a true directional anisotropy for auditory-to-visual changes in visual motion perception or is merely due to random sampling error.

In conclusion, whereas previous studies have failed to definitively establish whether apparent auditory motion can elicit a visual motion aftereffect, our results clearly demonstrate that exposure to horizontal auditory motion signals elicits a visual motion aftereffect. These findings are consistent with the maximum likelihood hypothesis of multisensory perception and support the notion that motion perception from multiple sensory inputs is governed by at least partially overlapping neural substrates.

## Author contributions

CB and HE designed the study. CB conducted the experiments and analyzed the data. CB drafted the manuscript, and HE provided critical revisions.

## Funding

This research was supported by the Riksbankens Jubileumsfond, the Swedish Research Council, and Torsten S6derbergs Stiftelse.

### Conflict of interest statement

The authors declare that the research was conducted in the absence of any commercial or financial relationships that could be construed as a potential conflict of interest.
